# The Perfect Surgeon

**Published:** 1907-02-23

**Authors:** 


### The Perfect Surgeon.

The Hunterian Oration delivered by Mr. Butlin at the Royal College of Surgeons of England on St. Valentine's Day—the anniversary of the birthday of John Hunter—was a model of what should be expected from a modern surgeon of cultivated tastes. It exemplifies at once the weakness and the strength of modern medical education. The weakness is slight and is easily dealt with, though it is common to the whole medical profession. It is neither more nor less than the neglect of medical history which leads the whole profession to ignore the history and work of their great predecessors. Nor have they the excuse alleged by Horace:

" Vixere fortes ante Agamemnona
Multi; sed omnes illacrymabiles
Urgentur, ignotique longâ
Nocte, carent quia vate sacro "

(many mighty ones lived before Agamemnon, but they are overwhelmed by everlasting night, unwept and unsung, because they had no sanctified bard). The Dictionary of National Biography contains an ample record of the work of our English surgeons, yet Mr. Butlin confesses frankly that he thought surgery before the time of Hunter was in a deplorable condition, and that he had forgotten Sharp and Pott. He might have added, too, Woodall and Wiseman, the great English surgeons of the Stuarts and the Commonwealth, surgeons who were thoughtful and clear writers, with a deep knowledge of the practical side of their profession. The view taken of the early surgeon is often that of John Earle, the Bishop of Salisbury, who says that a "surgeon differs from a physician as a sore does from a disease, or the sick from those that are not whole; the one distempers you within, the other blisters you without. The rareness of his custom makes him pitiless when it comes: and he holds a patient longer than our Courts a cause."

But great as were Hunter's predecessors, their surgery rested upon the frail foundation of anatomy alone. It was left to Hunter to point out that they who treat diseased bodies should have an intimate acquaintance with the structure and functions of those bodies in health, that they should know, so far as such knowledge is possible, the changes which those bodies undergo in disease and the further changes which they undergo in the progress towards recovery. He perceived that each part of the body must be studied not only by itself, but in relation to all other parts, and he came to the conclusion that great light might be thrown upon these matters by a study of similar parts in lower animals. For a knowledge of comparative anatomy would not only suggest the meaning of what otherwise might be regarded as meaningless or useless anomalies in man, but would furnish a much better criterion of the relative value of the various organs than could be acquired from their study solely in the human subject. It would be possible, too, to observe on animals the effects of injury and disease which had been deliberately produced, and perhaps to obtain a much clearer notion of the results of treatment than could be obtained in man. Such a course of self-training made Hunter a model surgeon. A perfect surgeon he never was, for to the end he remained rough and uncouth of speech, whilst his contempt for literature showed the lack of early education. But he can be forgiven these deficiencies, for, as Mr. Butlin well says, " The Huntarian oration ought to be delivered at one time by a naturalist, another time by an anatomist, again by a physiologist, and only from time to time by a surgeon. By putting together the lectures delivered by these various specialists, and in that way alone, could a just appreciation of John Hunter be obtained."

Even at the present day a surgeon might do much by educating himself on John Hunter's lines. Too often he is contented with so much biological knowledge as is required by the examining boards at the beginning of his career, whilst his experimental ardour and his zeal for original work is quickly strangled by the cares of many out-patients and much teaching. The time will shortly come when the young hospital surgeon will be relieved of both these trials. There is already evidence that medical students are taught so much that they have no time to think for themselves; and in the near future it is probable that the casualty patients who throng the hospitals will be more carefully sorted, and that their numbers will either be reduced very greatly or that they will wholly disappear, to the mutual advantage of the young surgeon who loses them and the poorer sort of medical practitioner who gains them as patients. The field of medical research is illimitable, and much of it is as yet untilled. Let us hope, therefore, that in the future original investigation of the true Hunterian type will again become a characteristic of the English school of surgeons, as it was at the beginning of the nineteenth century.

